# Easy Method for the Transformation of Levulinic Acid into Gamma-Valerolactone Using a Nickel Catalyst Derived from Nanocasted Nickel Oxide

**DOI:** 10.3390/ma12182918

**Published:** 2019-09-09

**Authors:** Rut Sanchis, Tomás García, Ana M. Dejoz, Isabel Vázquez, Francisco J. Llopis, Benjamín Solsona

**Affiliations:** 1 Departament d’Enginyeria Química, ETSE-UV, Universitat de València, Av. Universitat s/n, 46100 Burjassot, Valencia, Spain; rut.sanchis@uv.es (R.S.); ana.m.dejoz@uv.es (A.M.D.); isabel.vazquez@uv.es (I.V.); francisco.llopis@uv.es (F.J.L.); 2 Instituto de Carboquímica (ICB-CSIC), C/Miguel Luesma 4, 50018 Zaragoza, Spain; tomas@icb.csic.es

**Keywords:** levulinic acid, valerolactone, nickel, nanocasting, hydrothermal reaction, high temperature water

## Abstract

Different nickel catalysts have been tested for the transformation of levulinic acid into γ-valerolactone using an easy hydrothermal method, taking advantage of the properties of the high temperature water. A metallic nickel catalyst derived from NiO synthesized by a nanocasting procedure can achieve a productivity to γ-valerolactone, which is two orders of magnitude higher than that obtained by a commercial nickel catalyst. This nanocasted metallic nickel catalyst has shown bifunctionality as it is capable of activating water as the source for hydrogen and undertaking the further hydrogenation step. In contrast with metallic nickel, nickel oxide has shown to be incapable of transforming levulinic acid into γ-valerolactone.

## 1. Introduction

The restriction and/or ban in the use of diesel and petrol for transport is likely to take place before it was expected at the beginning of the century. In fact, in European countries, such as Norway, the UK, and Spain, the use of oil derivative fuels is projected to be banned by 2040. Different alternatives have been proposed, but those with the highest potential are electric vehicles (EVs), fuel cells vehicles, and the use of biofuels. Although bioethanol and biodiesel are widely employed biofuels, the use of cheap and abundant lignocellulosic biomass as a source for biofuels would mean a step forward in the progressive decarbonation of the automotive sector [1,2,3,4]. 

Among the compounds that can be obtained from the lignocellulosic biomass, we can highlight gamma-valerolactone (GVL). GVL can be obtained from the lignocellulosic biomass through the reduction and lactonization of levulinic acid (LA). LA is a fatty acid with a ketone and a carboxyl group which can be easily obtained from C6-sugars using acid catalysts (acidic hydrolysis) [5].

The transformation of LA into GVL can follow at least two pathways; firstly, LA can be hydrogenated to form a reaction intermediate, such as 4-hydroxypentanoic acid, or can be dehydrated to form anjelica lactone. Secondly, the intermediates can be cyclized/dehydrated, in the case of the 4-hydroxypentanoic acid, or reduced, in the case of anjelica lactone, to form the final GVL product [6,7].

GVL is a chemical compound with high energetic density that can be used as a precursor of clean fuels or, as such, as a component for advanced green fuels [8,9]. Interestingly, GVL can be easily and safely stored and transported, since it presents relatively low flammability and is a liquid in a wide range of temperatures (between −31 °C and 207 °C). In addition, GVL can be easily transformed into olefins and food additives or used directly as a solvent. 

The interest of the GVL is manifested by the high number of publications (172 for the past 10 years), of which around 30 are related to the transformation of LA into GVL using catalysts not derived from noble metal [10]. The selective transformation of LA into GVL can be undertaken by a number of methods, involving homogeneous and/or heterogeneous catalysts. Typical materials are based on noble metals [11,12,13,14,15,16,17] as they are those that achieve the highest productivity to GVL. Among noble metals, those of ruthenium seem to lead to the best results [18,19,20]. However, as noble metals are expensive, different options involving cheaper materials have been studied. Thus, supported metal catalysts [7,21] and mixed metal oxides [22,23], if properly optimized, have demonstrated to be almost as efficient as catalysts based on noble metals. Interestingly, a metal organic framework containing a zirconium catalyst has recently shown that using a continuous flow can transform methyl or ethyl levulinate into GVL. [24,25] 

Catalysts based on nickel have been reported for the LA transformation to the GVL reaction as they obtain yields comparable to those obtained with noble metals and also present the advantage of being easily recovered after reaction due to the magnetic characteristics of nickel catalysts. In most cases, nickel is supported on a range of materials [26,27,28,29,30], such as carbon, alumina, or magnesia.

This reaction requires reduction and dehydration steps, and therefore a source of hydrogen is necessary, molecular H_2_ being the most frequently used. Batch systems with H_2_ at high pressure are the most common, although there are several studies showing that gas-phase continuous systems working at a low temperature can also selectively produce GVL [31]. As the handling of H_2_ is not straightforward and the flammability is high, several alternatives have been proposed to avoid the use of molecular hydrogen. The use of formic acid as a hydrogen source has been studied [32,33], showing very reasonable GVL yields. Recently, it has been reported that 0 valence inexpensive metals, such as catalysts [34,35,36], can transform water into hydrogen by means of a simple hydrothermal method at high pressure and mild temperatures (ca. 250 °C). This in situ generated hydrogen can then be used for the hydrogenation of LA into GVL at the surface of the same metals [37]. Metallic nickel together with metallic iron have been proposed as the most efficient options.

In this article we have used this very simple hydrothermal method, in which water exerts as the reaction media and also as a source of hydrogen in order to achieve the LA transformation into GVL. This method is straightforward and does not require neither gaseous hydrogen nor expensive H-donors. Moreover, the catalysts to be employed are not based on expensive noble metals.

Thus, bulk metallic nickel catalysts have been tested. We have employed differently synthesized Ni based catalysts: (i) Commercial Ni metal, (ii) Ni obtained by the reduction of NiO from nickel nitrate, (iii) Ni derived from nickel oxide synthesized by a soft chemistry method with oxalic acid, and (iv) Ni derived from NiO synthesized by a nanocasting route. 

Importantly, the catalyst derived from metal oxides prepared by a nanocasting route has resulted to give the highest yields to GVL. 

## 2. Materials and Methods 

### 2.1. Materials 

#### 2.1.1. Synthesis of Nickel Catalysts

Commercial Ni was supplied by Alfa-Aesar–150–200mesh, Kalsruhe, Germany, 99.8%. This sample has been named NiCOM.

Standard nickel oxide was prepared by solving Ni(NO_3_)_2_·6H_2_O (Sigma–Aldrich, St. Louis, MO, USA, >98%) in ethanol. This solution was evaporated in a hot plate stirrer at 60 °C, dried in a furnace at 120 °C for 12 h, and then heat-treated in static air for 4 h at 500 °C. This NiO presents a surface area of 3.4 m^2^ g^−1^. Before the use in the reaction, this sample was heat-treated in flowing H_2_ for 3 h at 400 °C. This sample has been named NiSTD.

A third sample was synthesized evaporating an ethanolic solution of nickel (II) nitrate (Sigma–Aldrich, >98%) and oxalic acid (H_2_C_2_O_4_·2H_2_O, Sigma–Aldrich), being the molar Ni:oxalic ratio equal to 1:3. The evaporation of ethanol was carried out at 60 °C in a hot plate stirrer under vigorous stirring. The paste obtained was dried for 12 h at 120 °C and then heat-treated in static air in two steps; 2 h at 300 °C and 2 h at 500 °C. This nickel oxide presents a surface area of 15 m^2^ g^−1^. Previous to the reaction, this sample was heat-treated in flowing H_2_ for 3 h at 400 °C. This sample has been named NiOXA.

Nickel oxide was also prepared by a nanocasting method using a non-solvent impregnation route with KIT-6 as a siliceous hard template. In our synthesis, 30 mmol of Ni(NO_3_)_2_.6H_2_O (Sigma–Aldrich, >98%) was mixed with 0.45 g of mesoporous silica KIT-6 (see preparation below) and was ground for a few minutes in an agate mortar. The mixture was left in a crucible and placed in a muffle furnace. The temperature was increased from ambient temperature until 500 °C, using a rate of 1 °C min^−1^, and was kept at the final temperature for 5 h. The mesoporous metal oxide was recovered by mixing the previously calcined mixture with 50 mL of 2 M NaOH solution. This was then stirred at 80 °C in a hot plate stirrer for 24 h, centrifuged, washed with ethanol-distilled water mixture, and dried in a furnace at 120 °C overnight. The surface area of this nickel oxide was 39 m^2^ g^−1^. Before the reaction, this precursor was heat-treated in flowing H_2_ for 3 h at 400 °C. This sample has been named NiNC.

As mentioned above, KIT-6 is the mesoporous silica used as a hard template for the NiNC synthesis. KIT-6 mesoporous silica is a three-dimensional material with Ia3d cubic arrangement of pores. This pore structure achieves mesoporous morphologies with high thermal stability and highly crystalline walls. A KIT-6 template was prepared using acidic conditions with a mixture of butanol and Pluronic P123 triblock copolymer (EO_20_PO_70_EO_20_). Pluronic P123 (12 g) was added to a solution consisting of 440 g of deionized water and 24 g of HCl (35%). This mixture was vigorously stirred at 35 °C for 6 h, and then 12 g of butanol was added and left stirring for 1 more hour. A total of 24.96 g of tetraethyl orthosilicate (TEOS, 98%, Sigma–Aldrich) was then added at 100 °C for 24 h. The gel obtained was then submitted to a hydrothermal treatment (static conditions) in a stainless-steel autoclave, heating at 130 °C for 72 h. Finally, the solid recovered by filtration was washed with deionized water and dried for 24 h in a furnace. The final product was then calcined in static air at 550 °C for 6 h. 

#### 2.1.2. Characterization Techniques

Structural and morphological analyses of the catalysts were carried out by high resolution transmission electron microscopy (HRTEM) using a field emission gun TECNAI G2 F20 microscope (FEI Company, Hillsboro, OR, USA), operated at 200 kV. This equipment was also used for undertaking selected area electron diffraction (SAED) and energy dispersive X-ray spectroscopy (EDX). The preparation of the catalysts for the TEM analysis involved a treatment by sonicating the samples in absolute ethanol for several minutes. The suspension obtained was placed on a holey carbon film, which was supported on a copper grid. Finally, the copper grid was dried.

Catalysts were submitted to N_2_ adsorption at −196 °C, using a Micromeritics ASAP 2020 apparatus (Norcross, GA, USA). Catalysts were degassed at 150 °C before the analysis. Total pore volumes were determined, employing the adsorbed volume at a relative pressure of 0.97. A multipoint Brunauer–Emmet-Teller (BET) specific surface area (S_BET_) was determined through the relative pressure range from 0.05 to 0.25. The pore size distribution was analyzed using the Barrett–Joyner–Halenda (BJH) method by analyzing the adsorption branch of the N_2_ adsorption isotherms.

Powder X-ray diffraction (XRD) was employed to know the crystalline phases of the samples. An Enraf Nonius FR590 sealed tube diffractometer (Bruker, Delft, The Netherlands) with a monochromatic Cu Kα1 source operating at 40 kV and 30 mA was used. 

Temperature programmed reduction (TPR) experiments were conducted using a Micromeritics Autochem 2910 apparatus (Norcross, GA, USA) with a thermal conductivity detector. The gas employed consisted of a mixture of H_2_ in argon (10% hydrogen) with a total flow rate of 50 mL min^−1^. The temperature range studied was from ambient temperature to 700 °C, and the heating rate was fixed at 10 °C min^−1^.

#### 2.1.3. Catalytic Tests and Analyses

As mentioned above, after the metal oxide precursors had been prepared, they were reduced in a flow of H_2_ (30 mL min^−1^) for 3 h at 400 °C. The catalytic tests were undertaken using hydrothermal conditions, employing a 13 mL stainless steel autoclave, which was purged with N_2_ in order to minimize the possible metal oxidation. The autoclave inner walls were covered by a Teflon container (own manufacturing), which fits with the steel walls. The feed consisted of levulinic acid (140 mg) and water (3.5 mL). The amount of catalyst used was typically 62.5 mg. Other experiments with 20.8 and 125 mg were conducted in order to check the influence of the amount of catalyst on the catalytic performance. The autoclave was thoroughly sealed and was then introduced in an oven previously heated at the desired reaction temperature (mainly at 180 °C) for the duration of the experiments (typically 2 h), and it was then quickly cooled in an ice-bath. Reaction time was considered as the time the autoclave had been introduced in the oven (therefore there was an induction time until the mixture reached the desired temperature, which was considered in the reaction time). The product obtained was recovered by filtration using an appropriate membrane. 

For comparison, some selected standard experiments in the presence of hydrogen and stirring were conducted in a Parr autoclave (Moline, IL, USA). The batch reactor had a volume of 100 mL, whereas the stirring speed was fixed in 1200 rpm. Previous to the experiment, the batch was filled three times with hydrogen and vented. The pressure hydrogen employed was 3 MPa and the water volume was 40 mL. A total of 1 g of levulinic acid was used, and the amount of catalyst employed was 62.5 mg. After the reaction, the reaction mixture was cooled until room temperature with ice and then filtered, as in the former assays. 

### 2.2. Analytical Method

The products obtained were analyzed, as described in Reference [37]. The samples were identified by gas chromatography, using the GC instrument Hewlett Packard 5890 (Palo Alto, California, Estados Unidos), equipped with an Agilent HP-1 column (30 m × 0.32 mm × 0.25 µm). The detector employed was a FID detector working at 240 °C, and an injection port, working at 220 °C. The temperature program for a typical run was as follows: (i) 35 °C isothermal for 6 min, (ii) from 35 to 230 °C with a heating rate of 20 °C min^−1^, and (ii) isothermal at 230 °C for 26 min. The retention times for the main components GVL and LA were 5.4 and 9.5 min, respectively. The LA conversion was compared against a blank run, undertaken at room temperature and in absence of a catalyst. Experiments with a gas chromatography mass spectrometer (GC-MS 5977A MSD-7890A, Agilent, Santa Clara, CA, USA) were carried out in order to identify the minority reaction products.

## 3. Results and Discussion

### 3.1. Characterization Results

Table 1 shows some characteristics of the nickel oxide precursors and metallic nickel catalysts synthesized. The standard NiO precursor synthesized by a simple method (NiSTD precursor) presented the lowest surface area (3.4 m^2^ g^−1^). The sample prepared using oxalic acid (NiOXA precursor) showed an increased surface area, which was four times higher than that of the standard Ni. Finally, the sample prepared by the nanocasting route (NiNC precursor) reached the highest surface area, 39 m^2^ g^−1^. 

**Figure 1:**
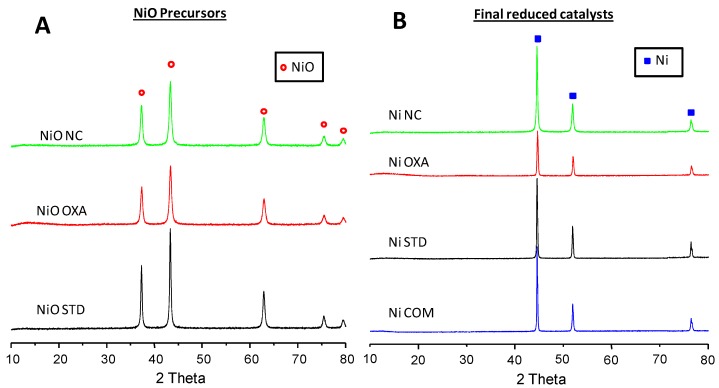
XRD patterns of NiO precursors (**A**) and final reduced Ni catalysts (**B**).

The XRD patterns of all the nickel oxide precursors (Figure 1) show that cubic NiO is the only crystalline phase detected (NiO, JCPDS: 47-1049). However, an appreciable difference can be observed in both the surface areas and the crystallite sizes (Table 1). The highest surface area (39 m^2^ g^−1^) and the lowest NiO crystallite size (19 nm) are then observed for the nanocasting sample. Conversely, the standard NiO presents the lowest surface area (3.4 m^2^ g^−1^) and the highest crystallite size (35 nm). As expected, an inverse relationship between surface area and NiO crystalline size is observed. 

Figure 1, also shows the XRD of the final reduced catalysts. It can be seen that the only crystalline phase present in these reduced catalysts is metallic Ni (JCPDS: 01-1260), although the width of the peaks slightly varies. As it has been observed for the precursors, the lowest surface area (<1 m^2^ g^−1^) and the highest mean Ni crystallite size (ca. 50 nm) correspond to the commercial catalyst. The surface area follows an inverse trend to that of the crystallite size. The surface area varies according to: NiCOM < NiSTD < NiOXA < NiNC, which is the same trend observed for the precursors. It must be noted that the surface areas of the precursors are in all cases higher than those of the reduced final catalysts. The additional heat treatment in hydrogen flow at 400 °C, with the consequent modification of the crystalline structure, are likely to be the responsible for this surface area drop. 

Low angle XRD from 2 theta = 1° was examined for both the NiO precursors, synthesized by a nanocasting route and the reduced final NiNC catalyst (not shown here). In the case of the final NiNC catalyst, no maxima associated to an ordered mesoporous structure were observed, whereas in the case of the precursor, the presence of low intensity bands at ca. 1.2 and 1.7° cannot be ruled out.

**Figure 2:**
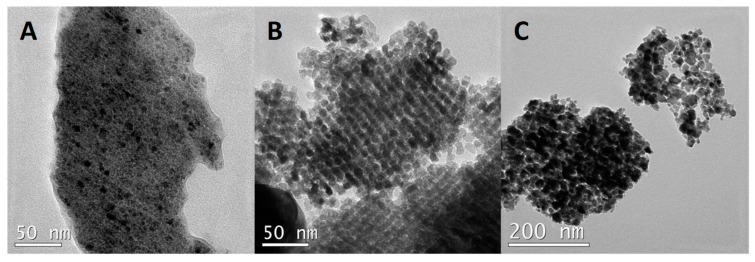
Representative transmission electron microscopy (TEM) images of different nickel containing samples: (**A**) NiCOM, (**B**) NiO precursor of NiNC, and (**C**) NiNC.

The precursor and the catalyst with the highest surface area are those prepared by a nanocasting route. This method allows high surface area materials with a high extent of order, and its shape is determined by the pores of the siliceous hard template used in the preparation method. In order to corroborate the appropriate preparation of this material, a TEM study was then undertaken. The NiO precursor shows the typical image of a nanocasted structure, with very well-defined rods and pores. Moreover, the use of KIT-6 allows the rods formed to be interconnected, making possible a compact structure (Figure 2). The length of the rods is quite homogeneous with a mean width of ca. 7 nm, whereas the free space between the rods is smaller than 1 nm wide (intraparticle porosity). Therefore, we can conclude that a reasonably good ordered replica of siliceous KIT-6 has been obtained.

Unfortunately, the reduction of the NiO prepared by nanocasting does not allow us to maintain the ordered structure, although the final catalyst keeps important porosity. NiNC presents a morphology consisting of agglomerations of nanoparticles (NPs). The agglomerations present a variable size; in fact, after a detailed study of the sample, we have observed relatively small agglomerations with diameters of 50 nm and some others exceeding 400 nm. The size of the nanoparticles conforming the agglomerations is mainly in the 6 to 20 nm range. Whilst intraparticle porosity completely disappears, some interparticle mesoporosity is preserved after H_2_ reduction. 

For comparison, the commercial metallic nickel (NiCOM) has been also studied by TEM. The morphology drastically changes compared to the former samples. Thus, large particles with sizes between 100 and 300 nm are predominant in this sample. Apparently, no porosity has been observed inside these particles. Interestingly, the image of the Ni particles detects zones with different contrast, although a detailed analysis by selected area electron diffraction (SAED) shows, for any zone of the catalyst studied, the unique presence of metallic Ni.

**Figure 3:**
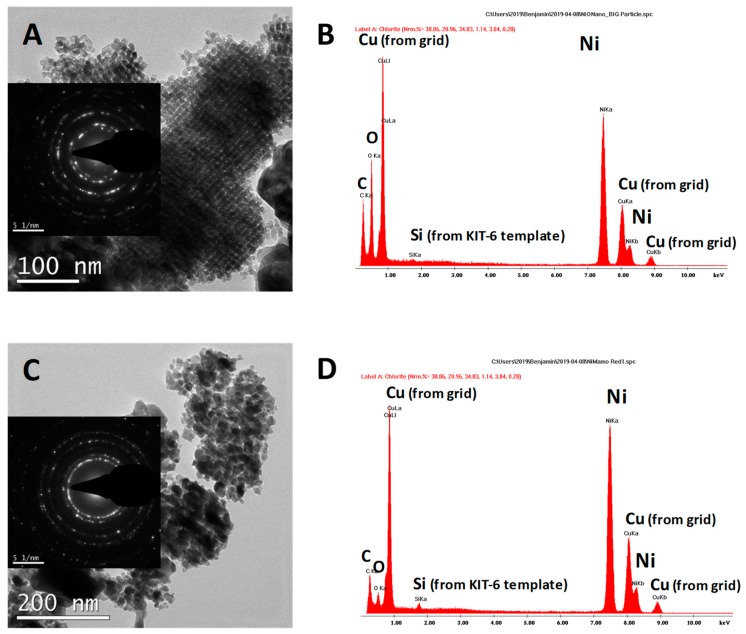
Representative TEM images with the corresponding selected area electron diffraction (SAED) patterns (inset) for (**A**) NiO precursor of NiNC and (**C**) NiNC catalyst. EDX analyses of (**B**) NiO precursor of NiNC and (**D**) NiNC catalyst.

Figure 3 shows the SAED patterns for both the precursor and the NiNC catalysts. The precursor shows six defined diffraction rings (inset of Figure 3A) that, in agreement with the NiO phase (JCPDS: 47-1049) and the Bragg diffraction law, can be indexed to a face-centered cubic phase of NiO. The main distances detected are 2.41, 2.09, 1.48, and 1.26 Å, which correspond to the (111), (200), (220), and (311) planes of cubic NiO. No other nickel containing phase has been detected along the catalyst.

In the case of the final NiNC catalyst, the diffraction rings show the only presence of metallic Ni, indicating that the hydrogen treatment submitted to the precursor has completely reduced the nickel oxide into metallic Ni. The main distances observed are 2.04, 1.78, 1.27, and 1.10 Å, which correspond to the (111), (200), (220), and (311) planes of cubic Ni. In this sample, no other Ni-phase has been detected. This is in agreement with the XRD observations. 

The nanocasting route for preparing metal oxides involves the elimination of the hard templates (in our case silica). Therefore, it is important to control the extent of the silica removal in order to check the quality of the synthesis. The chemical composition of the specimens was then undertaken by EDX analysis, using a transmission microscope. Thus, the EDX analyses of both precursor and NiNC catalysts are shown in Figure 3B,D. Positively, the amount of silicon observed in both cases is very low (Si/Ni at. ratio lower than 0.5%). In the EDX analyses, other elements, such as C and Cu, have been detected, due to the use as a sample holder of holey-carbon film supported on copper grids. Oxygen has been also detected, showing in the NiO precursor a higher relative amount than in the final catalyst, in agreement with the different nickel-containing species present in each sample. Moreover, in these samples, the presence of sodium from the sodium hydroxide used to remove silica can be possible if the washing procedure is not thoroughly undertaken. Interestingly, in our case, EDX analysis does not reveal the existence of sodium and, if present, is below the detection limit of the technique.

**Figure 4:**
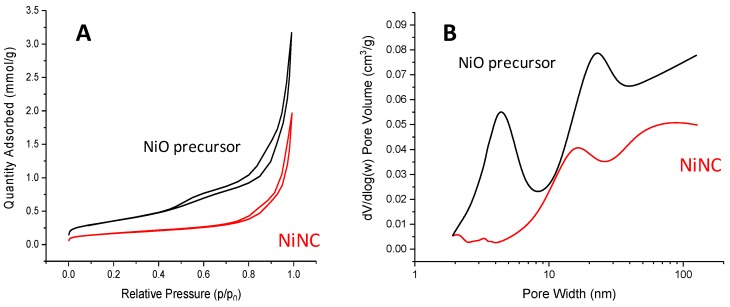
Adsorption-desorption isotherms (**A**) and pore distribution (**B**) of both the NiO precursor and the NiNC catalyst.

In order to know the surface area as well as the type of porosity for both the precursor and the NiNC catalyst, the adsorption-desorption isotherms and the pore distribution are shown in Figure 4. The NiO precursor synthesized by a nanocasting method presents a relatively high surface area, 39 m^2^ g^−1^, which is significantly decreased for the reduced sample, 13 m^2^ g^−1^. The nitrogen adsorption isotherms for the Ni-based catalysts prepared by the nanocasting method are presented in Figure 4A. Both isotherms are type IV with H1 type hysteresis, which are typical of mesoporous materials. It can be clearly observed that the NiO precursor accounts for both a more pronounced hysteresis and a higher N_2_ adsorption capacity at high relative pressures, again showing that the mesopore structure of the intraparticle porosity of the NiO partially collapses during the reduction step. The BJH approach applied to the adsorption branch of the isotherms is plotted in Figure 4B. For the NiO precursor and the NiNC catalyst, three different ranges of pores can be found, with mean pore sizes about 4 nm, 23 nm, and 10 nm, respectively. In a perfect KIT-6 replication process, the mesochannels of KIT-6 and their connecting microchannels are filled with the metal oxide, and then the siliceous walls between them are dissolved and eliminated, leading to the formation of pores of ca. 4 nm. However, if the partial replication process is not fully achieved, the simultaneous occupation of the KIT-6 microchannels is not accomplished and pores centered at about 15–20 nm are also obtained, which is consistent with observations in the current study. Moreover, it must be noted that for the NiO material there is some evidence of the formation of macropores (pores higher than 50 nm), likely proceeding from large interstitial pores generated between the NiO nanocast particles. The presence of this macroporosity could be positive, since it could improve the diffusion of large organic molecules to the active sites. A different pore size distribution is clearly observed after the reduction process. Whilst both broad mesopores at about 10–20 nm and macropores are significantly preserved, the narrowest mesopores observed for the NiO precursor at about 4 nm almost disappear. This evolution of the catalyst porosity after H_2_ reduction can be ascribed to the sintering due to the reduction treatment at high temperatures, as well as the modification of the structure due to the O-loss from the NiO precursor to the final Ni particles.

Therefore, a reasonable NiO replica of the siliceous KIT-6 has been obtained by the nanocasting route. Unfortunately, the reduction with hydrogen of NiO leads to the collapse of the pores at ca. 4 nm and a consequent drop in the surface area.

**Figure 5:**
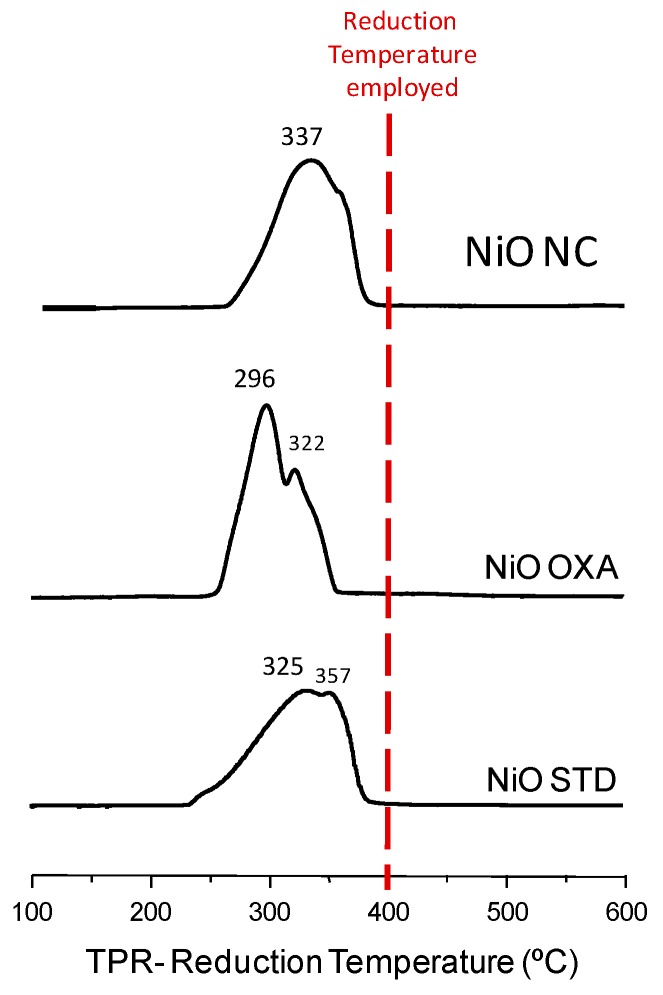
Temperature programmed reduction of nickel oxide precursors prepared by different preparation procedures.

Previously to undertaking the reaction, the nickel oxides were reduced at 400 °C in the hydrogen flow. We used that temperature to ensure that all nickel oxides were reduced into metallic nickel. The temperature programmed reduction experiments show that, for all nickel oxide precursors, total reduction takes place at temperatures below 400 °C (Figure 5). 

For all precursors, a main peak with a shoulder at higher temperatures is observed, which has been associated with larger NiO particles [38,39] due to certain heterogeneity in the samples. It must be noted that the hydrogen consumption in the TPR experiments adjusts with the NiO → Ni transition with a low error (±6%). 

### 3.2. Catalytic Results

In this work, we tried to take advantage of the properties of water at high temperature, since, this way, water can be the source for hydrogen, which is needed for the hydrogenation reaction of LA into GVL [37]. The typical reactions undertaken in this work are conducted using a closed autoclave with a teflon container at a reaction temperature of 180 °C. GVL is observed in most of the experiments conducted in the presence of catalysts. Conversely, the blank experiment in the absence of the catalyst gives a null conversion. In order to ensure the absence of catalytic potential of the bare reactor, experiments at higher temperatures were undertaken. Interestingly, at 220 °C, the LA conversion obtained without a catalyst is negligible.

**Figure 6:**
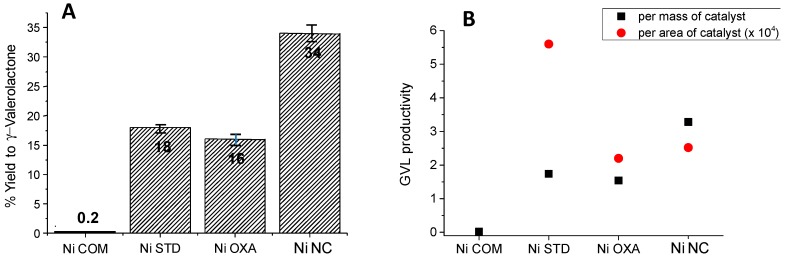
Catalytic results of the levulinic acid transformation into γ-valerolactone on different Ni catalysts: (**A**) Yield to γ-valerolactone, (**B**) productivity to γ-valerolactone per mass of catalyst (■) or per surface area (●). **Notes:** Reaction temperature = 180 °C, reaction time = 2 h, 62.5 mg of catalyst and remaining reaction conditions in the Materials and Method section. Productivity per mass of catalyst expressed as mol_GVL_ Kg_cat_^−1^ h^−1^. Productivity per area of catalyst expressed as 10^−4^ mol_GVL_ m^−2^ h^−1^.

The first experiments were undertaken at 180 °C with the differently prepared metallic Ni catalysts. Figure 6 shows the yield to GVL obtained by the different nickel catalysts. Commercial Ni showed a yield to GVL very low (ca. 0.2%). Significantly, the use of the other metallic nickel-based catalysts meant a drastic improvement. Using the standard nickel (NiSTD), a yield to GVL of 18% was then achieved. Similarly, the Ni catalyst synthesized from the NiO prepared with oxalic acid (NiOXA) achieved a yield of 16%. Finally, the Ni derived from the NiO prepared by a nanocasting route was the most efficient catalyst with a yield to GVL of 34%. It is interesting to note that the selectivity to GVL obtained by all the nickel catalysts exceeds 98% (Table 2). Apart from GVL traces of different by-products (formic acid, acetic acid, isobutyric acid, pentanoic acid, 4-hydroxy-2-butanone, and heavy oxygenated products) are observed.

The higher reactivity of the NiNC catalyst can be due to the higher surface area of this sample, this way allowing a higher number of active sites. However, if we consider the catalytic activity per surface area of the catalyst, we can observe that the sample NiSTD is the one with the highest value (5.6 × 10^−4^ mol_GVL_ m^−2^ h^−1^), whereas NiNC presents a lower value (2.52 × 10^−4^ mol_GVL_ m^−2^ h^−1^, respectively). The reason that explains this different areal rate is not straightforward to justify, although maybe a part of the lower areal rate of NiNC can be related to the presence of silicon on its surface, this way hindering the nickel-LA interaction. We have not given data of the areal rate obtained with the commercial metallic nickel because this catalyst presents a surface area which is lower than 1 m^2^ g^−1^, and then the error involved for determining the areal rate would be very high.

The catalytic performance of the NiO precursor in conventional reaction conditions was also explored. It has been widely reported that metals, such as Ni, are remarkably more efficient than their corresponding metal oxides. Accordingly, the experiment undertaken with the NiO precursor of NiNC demonstrates very low capacity for GVL production. In fact, not even traces of GVL have been observed in this case.

For a comparative purpose, these catalysts were tested in standard conditions in the presence of hydrogen (3 MPa), stirring (1200 rpm) at the same temperature of 180 °C. As the reaction conditions are not the same, see the Materials and Methods section, and an exact comparison is not possible. However, we have seen the same trend, using the hydrothermal conditions, as the nickel catalyst prepared by the nanocasting route was the most active and the commercial catalyst the least active. The selectivity observed in all cases was very high (over 95%). It is interesting to note that a yield of GVL higher than ca. 97% was obtained in the NiNC catalyst.

## 4. Discussion

**Figure 7:**
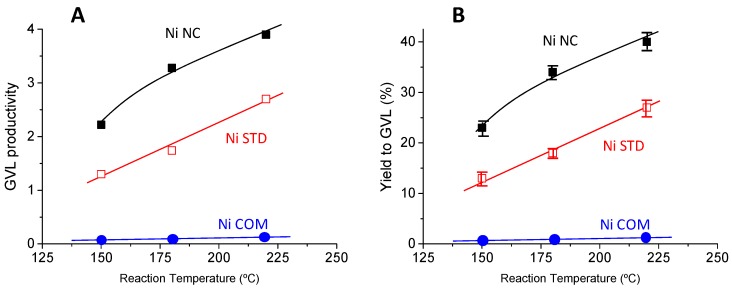
Effect of the reaction temperature on the catalytic performance of NiNC (■), NiSTD (□), and NiCOM (●): (**A**) Productivity to γ-valerolactone per mass of catalyst, (**B**) yield to γ-valerolactone, **Notes:** Reaction temperature = 150–220 °C, reaction time = 2 h, 62.5 mg of catalyst, remaining conditions in the Materials and Method section. Productivity per mass of catalyst expressed as mol_GVL_ Kg_cat_^−1^ h^−1^.

Once we knew that metallic nickel can activate levulinic acid at relatively low temperatures, we also wanted to determine the effect of the reaction conditions on the catalytic performance. Firstly, the influence of the reaction temperature on the LA conversion and on the productivity to GVL has been studied on NiCOM, NiSTDm and NiNC catalysts (Figure 7). As expected, the GVL formation increases with the reaction temperature in the temperature range studied (150–220 °C). Commercial nickel hardly forms GVL even at the highest temperature tested, whereas both NiSTD and NiNC present a similar trend, but with the catalyst derived from the nanocasting route presenting higher GVL formation.

**Figure 8:**
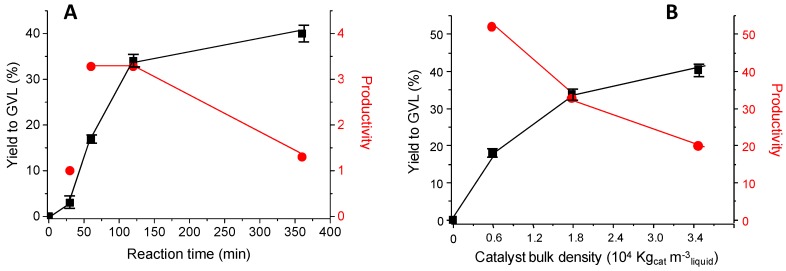
Influence of the reaction time (**A**) and the catalyst bulk density (**B**) on the yield to γ-valerolactone (■) and on the productivity to γ-valerolactone per mass of catalyst (●) using NiNC catalyst. **Notes:** Reaction temperature = 180 °C. (**A**) Catalyst weight = 62.5 mg and different reaction times. (**B**) Reaction time = 2 h and different catalyst bulk densities. Productivity per mass of catalyst expressed as mol_GVL_ Kg_cat_^−1^ h^−1^.

The influence of the reaction time on the catalytic performance has been also studied using the NiNC catalyst (Figure 8A). It can be observed that the yield to GVL increases when the reaction time increases. After 30 min in the furnace, the amount of GVL observed is not high (yield ca. 2%), whereas after 60 min and 120 min the yield increased to 17 and 34%, respectively. Further increase in the reaction time has led to an increase of the yield to GVL, although not proportional to the reaction time. If we consider the productivity (per unit time and per mass of catalyst) a maximum is observed at 60 and 120 min. The reason after the low productivity observed at 30 min can be due to the characteristics of the catalytic experiments carried out. In our tests, we place the autoclave into a furnace previously heated at 180 °C. For short reaction times, as in our case at 30 min, it was then possible that the temperature of the reaction mixture only reached the desired reaction temperature (180 °C) for a segment of that period. 

The effect of the amount of catalysts on the GVL formation has also been studied (Figure 8B). As expected, the higher the catalyst weight the higher the yield to GVL. However, the increase in the yield slows down when increasing the catalyst loading. Therefore, the productivity (per unit time and per mass of catalyst) decreases when the catalyst weight increases. 

In this article, it has been observed that there is no need to use noble metals in order to efficiently obtain GVL from LA under green and mild conditions. Moreover, the reaction method here employed is extremely easy. The results obtained indicate that metalllic Ni is highly active in the LA transformation into GVL. However, the preparation method of the Ni-based catalyst is of outstanding importance. Using the same reaction conditions, a commercial Ni then presents a GVL production of 0.019 mol_GVL_ Kg_cat_^−1^ h^−1^, whereas Ni derived from nanocasted NiO reaches a value which is two orders of magnitude higher (3.28 mol_GVL_ Kg_cat_^−1^ h^−1^). The higher productivity of NiNC could be related to its higher surface area and lower crystallite size, although the presence of silica on the surface as a result of the preparation method could have a negative impact. The role of the mesoporous structure cannot be elucidated, but it is clear that this approach involves an important catalytic improvement. 

In the reaction conditions employed in this article, the presence of the catalyst is essential as the lack of catalyst (mere contact with the Teflon container) does not lead to the LA transformation, even at 220 °C, which is 40 °C higher than the temperature employed in the present study. 

The characterization undertaken shows that the nickel catalysts only present metallic Ni, whereas no oxidized NiO is observed. TPR experiments corroborate that the reduction conditions employed are appropriate. The crystalline structure of the catalysts used in the reaction do not experience important differences compared to the unused catalysts, although some nickel oxide has been formed. Moreover, the estimated mean crystallite size hardly varies. A close look at the XRD pattern of the used NiNC catalyst shows that apart from the main peaks corresponding to metallic nickel and some low intense peaks that could be assigned to oxidized NiO are present. 

One of the main advantages of the use of supported nickel catalysts is their high reactivity, whereas one of the main drawbacks is associated with the lack of stability. The use of Ni-MoO_x_/C has been reported to lead to turnover frequencies similar to those obtained using catalysts with ruthenium [26]. Simple Ni/alumina catalysts achieved GVL yields over 90%, although, unfortunately, it was observed that the GVL formation importantly decreased after being reutilized [27]. Similarly, using Ni/MgO catalysts, high and unstable performance was reported [29]. Conversely, simple Ni/SiO_2_ catalysts showed a highly stable GVL production after 25 h of the experiment [28]. The use of dioxane as a solvent also had an important impact in the reusability of the catalysts [30]. Thus, the catalysts showed a stable activity after being used four times, in spite of the fact that nickel had partly oxidized into nickel oxide. Jiang et al. [40] observed that metallic nickel supported on MgO/Al_2_O_3_ was highly active at 160 °C, achieving GVL yields over 99.5%. This enhanced catalytic performance was related to the high dispersion of the nickel particles on the MgO/Al_2_O_3_ surface. These tiny metallic nickel particles were tightly interacting with the support, likely preventing leaching and allowing reutilization. In fact, no loss of activity for at least four uses was observed. 

The stability of the NiNC catalyst of the present article was also studied. In order to check the possible leaching in the reaction media, once the reaction finished, the catalyst was filtered. The liquid recovered was then submitted again to the standard reaction conditions (2 h at 180 °C) in the absence of the catalyst. After that time, it was observed that neither the LA conversion (35%) nor the yield to GVL 34%) increased compared to the initial test. In another set of experiments, the NiNC catalyst was reutilized in the reaction three times, but before its use the catalyst was pre-treated in hydrogen. In this case, it was observed that the catalyst did not deactivate as in the used catalyst and the activity obtained corresponded to the amount of catalysts employed, this suggesting that the active nickel sites fully recover its reactivity after reduction. Finally, if NiNC was re-used in the absence of pre-reduction, a decrease in the GVL yield was slight in the first three cycles, from 34% (first run) to 26% (third run), and then more drastic (10% in the fourth cycle). This fall can be ascribed to leaching of the active nickel in the reaction media and especially to the lack of hydrogen, due to the oxidation of metallic nickel. These data lead us to conclude that an industrial application using this system is difficult if the regeneration of the sample is not carried out in a simple manner.

**Figure 9:**
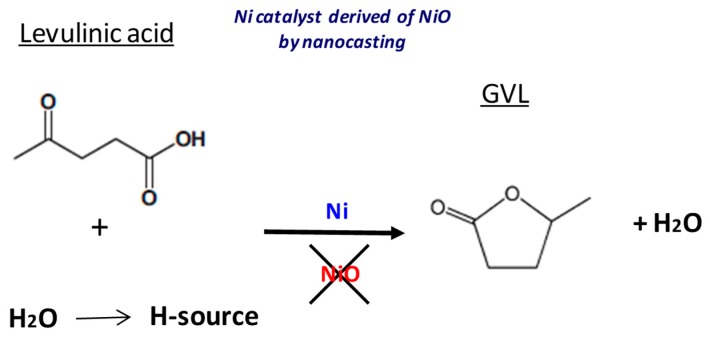
Scheme showing a summary of the experiments carried out in this work.

In the future, it would be very interesting to carry out a detailed mechanistic study on the GVL formation from LA using the easy reaction conditions employed in the present work. These metallic nickel catalysts, especially those prepared by a nanocasting route, have shown bifunctionality as they can activate water (as the source for hydrogen) and can also undertake the further hydrogenation step (Figure 9). Conversely to metallic nickel, any nickel oxide tested has shown to be incapable to transform levulinic acid into γ-valerolactone, indicating that nickel oxide cannot selectively activate levulinic acid in these reaction conditions. 

As mentioned above, levulinic acid transformation into GVL takes place by hydrogenation. The source for hydrogen atoms is molecular H_2_ in-situ formed. In several experiments, we have extracted the gas from the batch, and we have corroborated the H_2_ formation. Due to the reaction conditions employed, we have not been able to carry out a precise quantification of the amount of hydrogen formed, but we ensure that this has taken place as we have analyzed the gases after reaction. 

Water splitting to obtain hydrogen is easy even at low temperatures, employing a set of metals. By throwing selected metals in water, for example Mg, hydrogen can be directly obtained. However, this is not a catalytic process but the result of the oxidation of the metal to the corresponding oxide or hydroxide with the consequent water splitting and hydrogen formation. Unfortunately, most of metals in the earth are not in the metallic form but as oxides or salts, and then the reduction to the metallic form is not energetically favored. Metallic nickel sites are not only the active sites for the LA to GVL production, but also present the capacity for hydrogen production from water. In fact, nickel has been shown as highly active for water-to-hydrogen conversion via electro-oxidation [41,42]. The origin of the hydrogen proceeds from the water splitting as nickel metal plus water leads to the formation of metal oxide plus hydrogen (Ni + H_2_O → NiO + H_2_). Nickel the exerts both catalysts for the AL to GVL transformation and also as a source of hydrogen through its oxidation.

The hydrothermal method is a very straightforward reaction method and involves mixing the different participants in the reaction as water works at temperatures higher than that of its boiling point at ambient pressure. In these conditions, the vapor pressure of water increases, this way increasing the kinetic energy of the molecules and the rates of the possible reactions. Therefore, in these conditions there is a rather continuous contact between the reaction media and the catalyst. In fact, our experiments have shown to be highly reproducible. Unfortunately, there are possible mass transfer limitations due to the lack of stirring. In fact, according to our experiments, the variation of the GVL yield with the catalyst mass is not linear.

## 5. Conclusions

Metallic nickel derived from NiO synthesized by a nanocasting route presents a relatively high yield and selectivity to γ-valerolactone (up to 40% and >98%, respectively) in the transformation of levulinic acid, employing an easy hydrothermal method with water as a solvent at 180 °C. Through this method, the hydrogen proceeds from water and then hydrogenation of levulinic acid is possible. The productivity to γ-valerolactone reached using the nanocasted Ni catalyst is two orders of magnitude higher than that obtained by a commercial nickel catalyst. 

## Figures and Tables

**Table 1:** Characteristics of the nickel oxide precursors and final reduced nickel catalysts.

Preparation	Sample	S_BET_ (m^2^ g^−1^)	Crystalline Phases	NiO or Ni Crystallite Size by XRD (nm) ^1^	Residual Silicon Si/Ni at. Ratio % ^2^
Commercial	NiCOM	<1	Ni	47	~0
Standard	Precursor	3.4	NiO	35	~0
	Catalyst NiSTD	3	Ni	39	~0
Soft template	Precursor	15	NiO	26	~0
	Catalyst NiOXA	7	Ni	34	~0
Ordered, hard	Precursor	39	NiO	19	~0.3
Template	Catalyst NiNC	13	Ni	31	~0.4

^1^ Mean NiO crystallite size has been determined from the XRD patterns, applying the Scherrer equation; ^2^ amount of silicon detected by energy dispersive X-ray spectroscopy (EDX) analyses, expressed as Si/Ni at. ratio.

**Table 2:** Levulinic acid (LA) conversion, yield to GVL, and selectivity to GVL obtained at 180 °C using nickel catalysts. Comparison between the hydrothermal conditions mainly used in this work and standard conditions with hydrogen pressure and stirring.

Sample	Hydrothermal Conditions ^1^			Standard Conditions ^2^		
	Conversion	Yield	Selectivity	Conversion	Yield	Selectivity
NiCOM	0.2	0.2	100	7.9	7.4	95.2
NiSTD	17.9	17.7	98.8	79.2	77.2	97.5
NiOXA	16.7	16.4	98.2	75.7	74.3	98.2
NiNC	35.0	34.3	98.0	99.4	96.8	97.4

^1^ Reaction time = 2 h, 62.5 mg of catalyst, 0.14 mg of LA, 3.5 mL water; ^2^ reaction time = 2 h, 62.5 mg of catalyst, 1 g of LA, 1200 rpm, H_2_ pressure = 3 MPa.
